# Lactobacillus rhamnosus GG Evaluation in Acute Diarrhea (LEAD): An Observational Study

**DOI:** 10.7759/cureus.24594

**Published:** 2022-04-29

**Authors:** Mukesh Sanklecha, Lalit Verma, Uday Pai, Suman Mishra, Sameer Maqsood, Amita Birla

**Affiliations:** 1 Department of Pediatrics, Bombay Hospital Institute of Medical Sciences, Mumbai, IND; 2 Department of Pediatrics, SRCC Narayana Children’s Hospital, Mumbai, IND; 3 Pediatrics, Sai Kutir Clinic, Mumbai, IND; 4 Department of Pediatrics, Asian City Hospital and Sparsh Child Care, Patna, IND; 5 Department of Pediatrics, Max Super Speciality Hospital, Delhi, IND; 6 Clinical Research, ACT Life Sciences Pvt Ltd., Navi Mumbai, IND

**Keywords:** pediatric, loos stool, childhood diarrhea, bristol stool chart, acute gastroenteritis

## Abstract

Background and objective

Probiotics with documented efficacy such as *Lactobacillus rhamnosus* GG(LGG) and *Saccharomyces boulardii* might be utilized as adjuncts to rehydration for the management of acute gastroenteritis (AGE) in children. In this study, we aimed to evaluate the potential role of LGG in acute diarrhea in the Indian pediatric population.

Methods

An observational, cross-sectional study [*Lactobacillus rhamnosus* GG Evaluation in Acute Diarrhea (LEAD)] was conducted among children aged one month to 12 years with acute watery diarrhea. In addition to standard management of diarrhea, LGG was given as an adjuvant treatment at the discretion of treating physicians based on their routine practice. Observations were documented on days one, three, and five. Outcomes such as frequency and duration of diarrhea, time to change in consistency of stools, Bristol Stool Chart (BSC) reading, and global assessment by healthcare practitioners (HCPs) and patients were also recorded.

Results

Of the 2,080 patients enrolled, 1,900 completed the five-day follow-up. There was marked improvement observed in the number of incidences of loose stools from day one (mean: 7.33) to day five (mean: 1.6). The mean time to improvement in stool consistency was 34 hours. The mean duration of diarrhea was 44.63 hours. A relatively shorter duration of diarrhea was reported among participants in this study. There was also a significant improvement in the number of vomiting episodes. Most patients and HCPs reported the product to be excellent/very good on the global assessment scale. No adverse effects were noted in any of the groups.

Conclusion

Based on our findings, LGG supplementation may be a beneficial adjuvant treatment in reducing the severity and duration of acute diarrheal episodes.

## Introduction

Diarrhea is the third leading cause of childhood mortality in India and accounts for 13% of all deaths/year in children under five years of age [1]. Acute diarrhea is one of the most frequent gastrointestinal disorders and also the main cause of dehydration in childhood [2].

Acute diarrhea is usually self-limiting in children. However, appropriate treatment can considerably reduce the mortality rate. The treatment mainly entails the replacement of lost water and electrolytes and providing adequate nutrition. Probiotics are also helpful in managing childhood diarrhea. The use of antibiotics is justified only in certain cases of acute infectious diarrhea with positive stool culture for cholera, shigellosis, intestinal invasive amebiasis, giardiasis, or campylobacter infection [3].

Studies have shown that probiotics help to decrease the duration of diarrhea [4]. The rationale behind the use of probiotics in infectious diarrhea is that they compete with enteric pathogens for available nutrients and binding sites, and act against them. They also enhance specific and non-specific immune responses [5].

In 2008, the European Society for Pediatric Gastroenterology, Hepatology and Nutrition (ESPGHAN) and the European Society for Pediatric Infectious Diseases (ESPID) introduced evidence-based guidelines for the management of acute gastroenteritis (AGE) in children in Europe. These guidelines stated that probiotics with documented efficacy such as *Lactobacillus rhamnosus* GG (LGG) and *Saccharomyces boulardii* might be used as adjuncts to rehydration for the management of AGE in children [6]. There is an unmet need to ascertain the clinical outcomes of acute diarrhea in Indian children. This study titled *Lactobacillus rhamnosus* GG Evaluation in Acute Diarrhea (LEAD) was conducted across Indian cities to represent all zones of India. A clinical observation questionnaire was used in the study with a view to evaluating the potential role of LGG in acute diarrhea in the Indian pediatric population.

## Materials and methods

This was a cross-sectional observational study. Healthcare practitioners (HCPs) in pediatric clinics across India participated in the study. The study was conducted from June 1, 2021, to September 30, 2021. All children aged between one month and 12 years with the clinical diagnosis of acute diarrhea (less than seven days in duration) treated in the outpatient department (OPD) were eligible to be part of the study. The study was approved by the Institutional Ethical Committee (IEC) at the Dr. D. Y. Patil Medical College, Navi Mumbai. The need for informed consent from the parents of the patients was waived off by the IEC to avoid distress or confusion among participants and also to avoid biased outcome reporting.

The prescribing HCPs followed the standard-of-care management of diarrhea inclusive of but not limited to the administering of oral rehydration solution (ORS) and zinc for each patient. Participating HCPs were encouraged to add probiotics to the treatment, preferably LGG, considering the study's primary objective of LGG response data generation, and no other co-therapy. All participating HCPs were requested to prescribe Rhamo G (Alkem Laboratories Limited, Mumbai, India) to maintain uniformity in terms of medication. The prescription of LGG was at the discretion of treating physicians based on their routine practice, and data were collected only from those patients who received the prescription of LGG during the study period without adding risk to the patients. The anonymity of the patients was carefully ensured.

The participating HCPs were provided with a questionnaire through a password-protected online portal. The overall information regarding the approach to managing acute diarrhea and observational data focusing on the beneficial influence of LGG on the duration and severity of acute diarrhea in children were collected.

The observational data related to (1) number of incidences of loose stools, (2) consistency of the stool, (3) time since last loose stool in hours, (4) number of vomiting episodes, and (5) type of stool consistency graded as per Bristol Stool Chart (BSC) were collected on day one (baseline data), day three, and day five. The total duration of diarrhea and data related to the time to improve the stool consistency were also recorded on day five.

On day five, the overall observations were collected with the help of the global assessment on a 5-point scale (excellent, very good, good, fair, poor) by patients and the HCPs. The data were collected from patients to assess the parameters such as palatability, tolerability, ease of use, and overall outcomes. Data pertaining to adverse events were collected if any.

Statistical analysis

All the analyses were carried out using MedCalc Statistical Software version 19.0.6 (MedCalc Software bvba, Ostend, Belgium; http://www.medcalc.rorg; 2020). Quantitative data were presented as means with standard deviation (SD) and 95% confidence intervals (CI) and analyzed using one-way analysis of variance (ANOVA). Data for global assessments were presented as numbers with proportions. All analyses were done using two-sided tests with an alpha of 0.05. Missing data were not considered for analysis.

## Results

This observational pan-India study included 192 HCPs. The data were collected on 2,080 pediatric patients. Data were incomplete for 145 patients while a total of 35 patients were lost to follow-up. The data from 1,900 patients were considered for the analysis. Most of the patients were aged one to five years (61.3%), and 60% were male. In 33.7% of patients, the time since diarrhea was <12 hours, while it was 12-24 hours in 39.2%, and >24 hours in 27.2%.

The standard-of-care treatment with ORS and/or zinc along with LGG was given to 97.7% of patients. Only 2.3% of the patients received antibiotics, and ofloxacin was the most commonly used one. The most preferred dosing frequency was BID, followed by OD frequency, while TID was the least used dosing frequency. The most common total dose administered per day was 12 billion CFU of LGG prescribed among 1,333 patients, while six billion CFU of LGG was prescribed among 448 patients.

The mean duration of diarrhea (total duration from start to the stop of diarrhea) as recorded on day five was 44.63 hours. The number of incidences of loose stools decreased significantly from a mean of 7.73 on day one to 4.37 on day three (<0.0001) and 1.6 on day five (<0.0001) of treatment (Table 1). The mean time between the two consecutive stools also increased from 4.92 hours on day one to 8.64 hours on day three and 16.77 hours on day five of treatment.

**Table 1:** Improvement in diarrhea after treatment (n=1,900) SD: standard deviation; CI: confidence interval

	Observations (mean ± SD)	Change from day 1		P-value (versus day 1)
		Mean	95% CI	
No. of incidences of loose stools				
Day 1	7.73 ± 4.671	-	-	-
Day 3	4.37 ± 13.076	3.359	2.76–3.96	<0.0001
Day 5	1.60 ± 2.462	6.129	5.92–6.34	<0.0001
Bristol Stool Chart score				
Day 1	6.46 ± 1.08	-	-	-
Day 3	4.83 ± 1.43	1.629	1.56–1.69	<0.0001
Day 5	3.52 ± 1.43	2.937	2.86–3.01	<0.0001

An improvement in stool consistency was seen post-treatment. On day one, the patients had a BSC mean score of 6.46, confirming that they were suffering from diarrhea. The BSC score improved significantly post-treatment on day three (p<0.0001) and day five (p<0.0001), with a mean score of 3.52 on day five (Table 1). The mean time to improvement in stool consistency was 34 hours.

A total of 56.3% of patients had at least one episode of vomiting. The mean episodes of vomiting significantly (p<0.0001) decreased from 1.48 on day one to 0.2 on day five (Table 2).

**Table 2:** Improvement in vomiting post-treatment (n=1,900) SD: standard deviation; CI: confidence interval

	No. of vomiting episodes (mean ± SD)	Change from day 1		P-value (versus day 1)
		Mean	95% CI	
Day 1	1.48 ± 1.740	-	-	-
Day 3	0.46 ± 1.009	1.024	0.96–1.09	<0.0001
Day 5	0.20 ± 0.880	1.286	1.21–1.37	<0.0001

**Figure 1:**
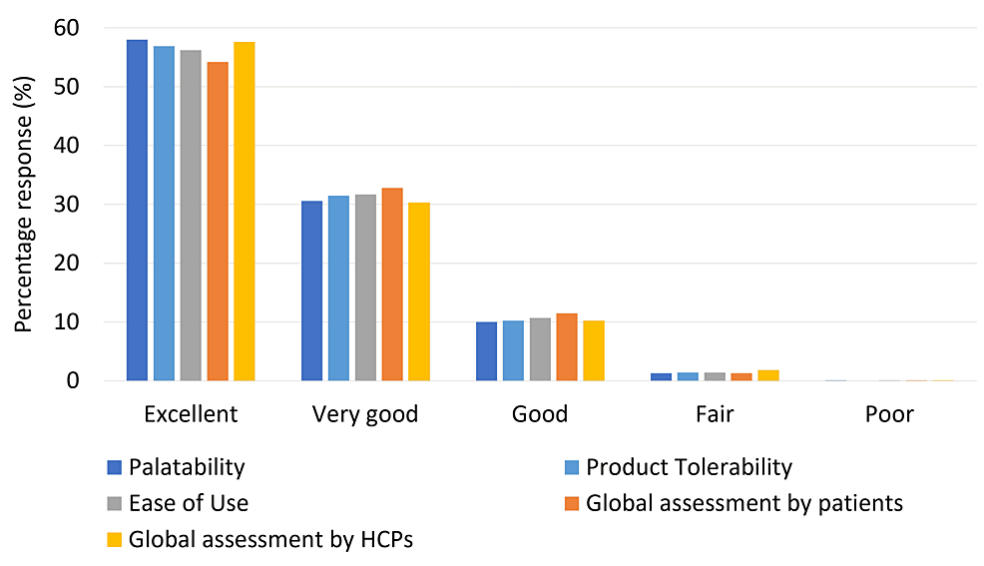
Global assessment of the product by patients and healthcare professionals (HCPs)

Most patients and HCPs reported the product to be excellent/very good on the global assessment scale (Figure 1).

## Discussion

The use of probiotic microorganisms is a proven intervention capable of reducing the risks of diarrhea in the early years of life. LGG has been one of the most widely studied probiotic strains, used in various commercially available probiotic formulations [7]. Probiotics are generally considered to be safe and easily administered [8].

The LEAD study compiled the observational data on the use of and therapy response to LGG among Indian pediatric patients with acute diarrhea. The LGG supplementation limited the extent of diarrheal symptoms during episodes of acute diarrhea with marked improvement in various symptomatic and clinical parameters. The beneficial effects of LGG were irrespective of the cause of diarrhea and it was reasonable to assume that most of the children were affected by acute viral gastroenteritis.

The use of LGG for the reduction of the duration of diarrhea is supported by convincing evidence [9]. A review of randomized clinical trials (RCTs) on LGG in the treatment of AGE in children has reported a reduced duration of diarrhea at a daily dose of ≥10^10^ CFU or ≤10^10 ^CFU by approximately 20 hours [10]. In infants and young children, the duration of a diarrheal episode was reported to be as long as three days [11]. A meta-analysis evaluating LGG in children aged 1-36 months reported that LGG was associated with a significant reduction in the duration of diarrhea [12]. Duration of diarrhea in hours is an important outcome from a clinical point of view [13]. Another trial evaluating LGG in the treatment of acute childhood diarrhea in India has reported a shorter duration of diarrhea with LGG [60 hours (54-72) vs. 78 hours (72-90); p<0.001] with faster improvement in stool consistency in children receiving LGG than the control group [36 (30-36) h vs 42 (36-48) h; p<0.001] [14]. The outcomes in the LEAD study were comparable with these reports. The mean time to improvement in stool consistency was 34 hours and the mean duration of diarrhea was 44.63 hours. A relatively shorter duration of diarrhea was reported in this study.

In a systematic review with meta-analysis, reduced duration of diarrhea [mean difference (MD): -24.02 h] was reported after the LGG administration [15]. High-dose LGG also effectively reduced the duration of rotavirus-induced diarrhea (MD: -31.05 h) and the stool number per day (MD: -1.08, 95%) [15]. Even in the present study, the number of incidences of loose stools reduced significantly on day three (4.37) and day five (1.60) from 7.73 on day one. In the LEAD study, significant improvement in vomiting episodes was also noted.

Long-term safety assessment in children who received LGG in a five-year follow-up study showed that LGG was associated with normal growth and development and long-term safety [16]. There were no adverse events reported in our study, and the product was well tolerated overall and graded as an easy-to-use formulation by the patients' parents or caregivers.

The promising results of the LEAD study share a similar trend with published literature and endorse the use of LGG co-supplementation in the management of acute diarrhea in children. The LEAD study has provided real-world evidence for the efficacy of co-supplementation of LGG, especially in the Indian setting. The reduction in the total time of diarrhea, number of incidences of loose stools, and improvement in stool consistency significantly impact patient care, especially for the parents. Also, this particular study demonstrated minimal rationale for the use of antibiotics, which is reassuring.

This study has a few limitations. Primarily, it was a clinical questionnaire-based study. The data recorded was as per details given by the parents and caregivers, which is liable to some subjectivity. The data was collected among OPD patients only, and hence was limited by constraints such as the inability to assess the 24-hour stool output. No specific investigations were done into the cause and diagnosis of acute diarrhea. Even though our study has the inherent limitations of an observational study, it highlights the potential role of LGG as an adjuvant in the management of childhood diarrhea, which seems to be a promising finding that can have a significant impact on patient care.

## Conclusions

Probiotics have been proven to be effective in reducing the risks of diarrhea in children. LGG is one of the most widely evaluated probiotic strains. It has also received recommendations in pediatric settings for the management of AGE.

In the LEAD study, physicians' opinions based on clinical observations demonstrated marked improvement in symptomatic and clinical parameters with LGG supplementation in children with acute diarrhea. The co-supplementation of LGG to the standard-of-care treatment can be a beneficial adjuvant in reducing the severity and duration of acute diarrheal episodes in the management of acute diarrhea among pediatric patients.
